# Supplementary material for: A Likelihood Approach for Real-Time Calibration of Stochastic Compartmental Epidemic Models
Source: PLoS Comput Biol. 2017 Jan 17;13(1):e1005257. doi: 10.1371/journal.pcbi.1005257 (PMC5240920; doi:10.1371/journal.pcbi.1005257)
Supplement: S1 File — (TAR.GZ) [file pcbi.1005257.s014.tar.gz › HSPH_Online-SI-Revision/EnKF07/n1-severe/n1-severe_EnKF2_table-8.pdf]

1	newly 3 weeks spec	19.	30.24	{2., 66.}	135.158	0.506999	newly 3 weeks cumul	34.	227.12	{37., 491.}	573.706	0.634146	newly 5 weeks spec	25.	9.81	{0., 21.}	88.36	1.28704	newly 5 weeks cumul	86.	252.21	{46., 624.}	212.733	0.373831	newly 62 weeks cumul	592.	275.8	{50., 701.}	73.7466	0.618889
2	newly 3 weeks spec	7.	53.06	{3., 113.}	680.857	0.831855	newly 3 weeks cumul	19.	370.36	{135., 841.}	1852.21	1.20184	newly 5 weeks spec	33.	17.99	{0., 43.}	72.7576	0.961157	newly 5 weeks cumul	67.	418.61	{166., 930.}	530.433	0.746414	newly 64 weeks cumul	626.	444.01	{171., 996.}	49.9569	0.350538
3	newly 3 weeks spec	17.	44.85	{2., 86.}	205.118	0.531122	newly 3 weeks cumul	50.	312.04	{77., 774.}	529.96	0.66716	newly 5 weeks spec	23.	14.03	{0., 36.}	87.1739	0.995618	newly 5 weeks cumul	88.	350.26	{96., 862.}	306.182	0.497222	newly 63 weeks cumul	618.	374.45	{113., 890.}	64.2379	0.451719
4	newly 3 weeks spec	5.	87.62	{9., 209.}	1660.4	1.09579	newly 3 weeks cumul	13.	482.03	{176., 902.}	3610.23	1.45385	newly 5 weeks spec	23.	36.95	{0., 91.}	123.435	0.701251	newly 5 weeks cumul	59.	575.25	{233., 1045.}	880.559	0.929588	newly 66 weeks cumul	656.	661.33	{280., 1126.}	46.8277	0.25834
5	newly 3 weeks spec	21.	53.41	{6., 109.}	184.048	0.468276	newly 3 weeks cumul	47.	520.47	{175., 977.}	1009.6	0.942288	newly 5 weeks spec	31.	16.83	{0., 46.}	82.6129	1.07746	newly 5 weeks cumul	110.	565.42	{237., 1060.}	418.455	0.640554	newly 65 weeks cumul	613.	599.94	{244., 1078.}	49.5106	0.254976
6	newly 3 weeks spec	13.	24.19	{2., 60.}	143.615	0.512291	newly 3 weeks cumul	22.	196.63	{38., 376.}	796.955	0.775226	newly 5 weeks spec	15.	9.36	{0., 29.}	99.7333	1.06402	newly 5 weeks cumul	45.	219.85	{48., 403.}	395.356	0.543618	newly 62 weeks cumul	650.	254.02	{48., 664.}	72.5538	0.659328
7	newly 3 weeks spec	23.	28.54	{2., 60.}	101.13	0.457933	newly 3 weeks cumul	69.	205.24	{36., 474.}	216.	0.391408	newly 5 weeks spec	34.	10.61	{0., 24.}	89.6176	1.32369	newly 5 weeks cumul	132.	231.6	{38., 586.}	114.758	0.31682	newly 62 weeks cumul	490.	259.53	{39., 681.}	72.2673	0.576738
8	newly 3 weeks spec	11.	87.23	{8., 212.}	707.182	0.796207	newly 3 weeks cumul	28.	498.12	{158., 946.}	1683.64	1.16236	newly 5 weeks spec	39.	36.73	{0., 84.}	72.3846	0.737756	newly 5 weeks cumul	95.	590.65	{225., 1051.}	528.158	0.759256	newly 66 weeks cumul	644.	673.42	{283., 1121.}	47.6087	0.265482
9	newly 3 weeks spec	13.	35.59	{2., 87.}	226.538	0.53164	newly 3 weeks cumul	27.	231.79	{35., 617.}	763.815	0.740979	newly 5 weeks spec	24.	13.45	{0., 36.}	92.5417	1.13972	newly 5 weeks cumul	73.	265.98	{38., 673.}	279.918	0.448847	newly 62 weeks cumul	503.	300.01	{42., 765.}	69.493	0.533541
10	newly 3 weeks spec	19.	30.1	{2., 65.}	124.842	0.483333	newly 3 weeks cumul	44.	225.06	{56., 529.}	416.591	0.555964	newly 5 weeks spec	47.	9.5	{0., 24.}	86.1277	1.3549	newly 5 weeks cumul	144.	249.99	{63., 629.}	107.507	0.283325	newly 62 weeks cumul	637.	272.62	{63., 684.}	68.7661	0.586052
11	newly 3 weeks spec	13.	18.1	{0., 33.}	156.154	0.823802	newly 3 weeks cumul	42.	122.34	{6., 235.}	245.667	0.441508	newly 5 weeks spec	27.	4.05	{0., 11.}	89.8889	1.68664	newly 5 weeks cumul	89.	134.96	{6., 342.}	151.034	0.579964	newly 59 weeks cumul	567.	140.56	{6., 467.}	88.2363	1.16152
12	newly 3 weeks spec	17.	86.59	{2., 196.}	437.706	0.772472	newly 3 weeks cumul	30.	442.74	{103., 881.}	1379.27	1.07114	newly 5 weeks spec	56.	44.51	{0., 116.}	67.4821	0.781885	newly 5 weeks cumul	124.	550.36	{128., 968.}	352.484	0.62084	newly 67 weeks cumul	637.	660.89	{169., 1074.}	42.416	0.245451
13	newly 3 weeks spec	11.	24.55	{2., 52.}	173.545	0.456123	newly 3 weeks cumul	31.	183.97	{34., 320.}	498.677	0.609649	newly 5 weeks spec	30.	8.79	{0., 22.}	85.9667	1.25812	newly 5 weeks cumul	95.	206.36	{38., 362.}	145.053	0.333312	newly 62 weeks cumul	650.	231.65	{38., 474.}	73.2662	0.686863
14	newly 3 weeks spec	13.	86.01	{3., 194.}	589.923	0.813063	newly 3 weeks cumul	26.	451.54	{94., 965.}	1639.92	1.13379	newly 5 weeks spec	24.	44.03	{0., 117.}	153.708	0.875706	newly 5 weeks cumul	70.	558.93	{117., 977.}	704.757	0.846537	newly 67 weeks cumul	660.	662.35	{145., 1058.}	41.7167	0.262046
15	newly 3 weeks spec	34.	10.32	{0., 20.}	82.4706	1.17772	newly 3 weeks cumul	78.	147.41	{23., 334.}	122.885	0.311725	newly 5 weeks spec	40.	2.14	{0., 5.}	94.65	1.99031	newly 5 weeks cumul	154.	153.92	{25., 336.}	78.4416	0.379995	newly 61 weeks cumul	487.	157.28	{25., 336.}	78.3901	0.758847
16	newly 3 weeks spec	11.	89.46	{4., 189.}	734.909	0.87479	newly 3 weeks cumul	21.	431.34	{91., 829.}	1958.1	1.19801	newly 5 weeks spec	31.	47.65	{0., 118.}	121.258	0.744578	newly 5 weeks cumul	74.	546.95	{114., 930.}	645.581	0.805259	newly 67 weeks cumul	620.	660.78	{220., 1051.}	41.8323	0.228865
17	newly 3 weeks spec	34.	84.06	{2., 184.}	191.294	0.639225	newly 3 weeks cumul	57.	422.45	{59., 832.}	649.877	0.794431	newly 5 weeks spec	41.	44.72	{0., 118.}	86.1951	0.763314	newly 5 weeks cumul	126.	530.48	{112., 919.}	332.413	0.598416	newly 67 weeks cumul	668.	643.47	{175., 1042.}	38.8458	0.242781
18	newly 3 weeks spec	10.	84.88	{3., 192.}	774.4	0.886881	newly 3 weeks cumul	22.	446.43	{96., 917.}	1932.5	1.19806	newly 5 weeks spec	22.	44.2	{0., 118.}	161.273	0.800051	newly 5 weeks cumul	59.	553.11	{117., 968.}	841.034	0.900904	newly 67 weeks cumul	630.	657.95	{201., 1051.}	43.6302	0.250014
19	newly 3 weeks spec	31.	20.39	{0., 36.}	108.032	1.14107	newly 3 weeks cumul	69.	153.85	{18., 468.}	173.58	0.36458	newly 5 weeks spec	48.	9.1	{0., 23.}	95.7917	1.83635	newly 5 weeks cumul	148.	176.67	{18., 705.}	116.48	0.509646	newly 60 weeks cumul	633.	196.38	{18., 882.}	81.3239	0.928865
20	newly 3 weeks spec	33.	17.49	{0., 41.}	99.7879	1.0899	newly 3 weeks cumul	87.	153.98	{24., 530.}	146.644	0.394338	newly 5 weeks spec	54.	6.08	{0., 14.}	90.2963	1.89907	newly 5 weeks cumul	182.	170.06	{27., 530.}	103.582	0.557106	newly 60 weeks cumul	622.	184.37	{34., 591.}	81.545	0.920397
21	newly 3 weeks spec	27.	88.89	{2., 195.}	270.926	0.70813	newly 3 weeks cumul	47.	434.66	{47., 876.}	831.574	0.865753	newly 5 weeks spec	26.	44.57	{0., 111.}	141.269	0.82446	newly 5 weeks cumul	97.	545.36	{87., 898.}	471.835	0.696964	newly 67 weeks cumul	609.	655.05	{167., 1049.}	44.1494	0.248716
22	newly 3 weeks spec	20.	9.41	{0., 24.}	73.85	0.840875	newly 3 weeks cumul	51.	134.92	{21., 315.}	191.961	0.404091	newly 5 weeks spec	65.	3.43	{0., 6.}	96.2615	2.13407	newly 5 weeks cumul	155.	143.15	{21., 326.}	71.0516	0.386525	newly 61 weeks cumul	627.	149.15	{21., 375.}	77.8389	0.845001
23	newly 3 weeks spec	22.	41.74	{2., 71.}	142.727	0.537026	newly 3 weeks cumul	61.	316.32	{79., 778.}	424.426	0.60233	newly 5 weeks spec	31.	12.2	{0., 30.}	76.7097	1.06783	newly 5 weeks cumul	113.	351.04	{99., 817.}	221.15	0.418526	newly 63 weeks cumul	543.	370.28	{105., 871.}	62.6188	0.417634
24	newly 3 weeks spec	19.	27.28	{0., 99.}	161.684	0.903944	newly 3 weeks cumul	60.	177.85	{20., 586.}	235.75	0.393726	newly 5 weeks spec	37.	11.04	{0., 35.}	97.5676	1.7452	newly 5 weeks cumul	115.	206.02	{22., 805.}	158.539	0.482583	newly 60 weeks cumul	485.	225.36	{23., 920.}	84.932	0.825946
25	newly 3 weeks spec	31.	8.01	{0., 11.}	100.742	1.69072	newly 3 weeks cumul	67.	94.99	{6., 93.}	131.806	0.520512	newly 5 weeks spec	70.	2.61	{0., 5.}	97.0714	2.49005	newly 5 weeks cumul	176.	102.35	{6., 97.}	102.119	0.837847	newly 58 weeks cumul	653.	107.21	{6., 97.}	90.2466	1.30713
26	newly 3 weeks spec	28.	52.38	{4., 112.}	125.929	0.432746	newly 3 weeks cumul	57.	519.12	{178., 983.}	813.825	0.863394	newly 5 weeks spec	43.	16.62	{0., 44.}	78.9767	1.23255	newly 5 weeks cumul	130.	563.18	{233., 1053.}	339.123	0.577276	newly 65 weeks cumul	587.	598.84	{234., 1075.}	50.6542	0.252198
27	newly 3 weeks spec	19.	55.55	{3., 122.}	221.316	0.545503	newly 3 weeks cumul	36.	372.76	{138., 891.}	938.944	0.925629	newly 5 weeks spec	37.	17.44	{0., 43.}	70.8649	0.894832	newly 5 weeks cumul	94.	421.9	{150., 966.}	353.83	0.590352	newly 64 weeks cumul	546.	448.46	{157., 1052.}	49.2271	0.288807
28	newly 3 weeks spec	3.	83.3	{3., 191.}	2688.67	1.24349	newly 3 weeks cumul	9.	447.22	{89., 951.}	4870.67	1.56114	newly 5 weeks spec	27.	44.92	{0., 122.}	137.481	0.820931	newly 5 weeks cumul	65.	556.26	{117., 962.}	760.831	0.854285	newly 67 weeks cumul	649.	660.61	{164., 1054.}	41.869	0.236845
29	newly 3 weeks spec	26.	18.2	{0., 41.}	108.692	1.06721	newly 3 weeks cumul	77.	157.67	{20., 539.}	162.299	0.378798	newly 5 weeks spec	43.	7.44	{0., 21.}	92.6977	1.85654	newly 5 weeks cumul	154.	175.86	{22., 676.}	114.662	0.524679	newly 60 weeks cumul	551.	188.25	{23., 822.}	82.7423	0.893572
30	newly 3 weeks spec	38.	9.86	{0., 19.}	82.3158	1.14652	newly 3 weeks cumul	100.	121.01	{15., 164.}	79.59	0.375374	newly 5 weeks spec	52.	2.17	{0., 5.}	95.8269	2.04914	newly 5 weeks cumul	190.	127.75	{15., 204.}	70.5842	0.5207	newly 61 weeks cumul	601.	130.86	{15., 237.}	81.0549	0.931227
31	newly 3 weeks spec	14.	28.61	{2., 54.}	164.5	0.532171	newly 3 weeks cumul	56.	194.95	{29., 419.}	268.268	0.458035	newly 5 weeks spec	13.	9.77	{0., 22.}	103.154	1.00487	newly 5 weeks cumul	98.	220.76	{32., 467.}	158.224	0.372605	newly 62 weeks cumul	505.	247.49	{32., 602.}	73.5545	0.630446
32	newly 3 weeks spec	13.	53.86	{3., 154.}	349.231	0.660669	newly 3 weeks cumul	24.	365.64	{142., 815.}	1424.67	1.08278	newly 5 weeks spec	8.	17.85	{0., 45.}	192.875	0.835968	newly 5 weeks cumul	57.	415.1	{155., 910.}	631.158	0.780072	newly 64 weeks cumul	509.	440.82	{165., 987.}	48.4597	0.273267
33	newly 3 weeks spec	19.	89.23	{2., 182.}	399.632	0.764092	newly 3 weeks cumul	30.	428.39	{88., 837.}	1332.77	1.05087	newly 5 weeks spec	36.	46.88	{0., 121.}	98.1667	0.722139	newly 5 weeks cumul	97.	540.16	{124., 909.}	465.546	0.702338	newly 67 weeks cumul	689.	658.76	{215., 1060.}	37.3295	0.231894
34	newly 3 weeks spec	23.	11.31	{0., 12.}	98.4783	1.01976	newly 3 weeks cumul	76.	96.92	{7., 123.}	121.105	0.529066	newly 5 weeks spec	29.	4.16	{0., 7.}	94.5517	1.77541	newly 5 weeks cumul	132.	107.49	{10., 144.}	102.932	0.669611	newly 59 weeks cumul	459.	114.8	{10., 211.}	88.2092	1.09656
35	newly 3 weeks spec	15.	88.08	{2., 192.}	519.067	0.823577	newly 3 weeks cumul	32.	435.74	{51., 846.}	1266.31	1.01946	newly 5 weeks spec	45.	44.1	{0., 117.}	81.6444	0.817047	newly 5 weeks cumul	99.	543.72	{83., 913.}	459.919	0.694612	newly 67 weeks cumul	642.	648.78	{153., 1033.}	41.0779	0.254842
36	newly 3 weeks spec	67.	43.67	{1., 115.}	70.0448	0.716394	newly 3 weeks cumul	106.	308.32	{19., 818.}	229.698	0.512101	newly 5 weeks spec	61.	29.8	{0., 99.}	79.3115	1.29907	newly 5 weeks cumul	222.	372.07	{42., 826.}	118.689	0.463817	newly 68 weeks cumul	649.	500.99	{42., 916.}	48.2388	0.447416
37	newly 3 weeks spec	27.	8.55	{0., 14.}	97.0741	1.52599	newly 3 weeks cumul	77.	87.44	{5., 118.}	119.325	0.581026	newly 5 weeks spec	49.	2.74	{0., 5.}	96.6531	2.19388	newly 5 weeks cumul	165.	94.38	{5., 120.}	99.0303	0.817266	newly 58 weeks cumul	598.	98.12	{5., 125.}	90.5886	1.2881
38	newly 3 weeks spec	10.	101.	{11., 218.}	921.6	0.907724	newly 3 weeks cumul	38.	492.36	{179., 886.}	1200.95	1.03113	newly 5 weeks spec	51.	43.58	{0., 91.}	62.1961	0.630738	newly 5 weeks cumul	123.	600.14	{239., 1053.}	395.366	0.658077	newly 66 weeks cumul	597.	691.06	{299., 1134.}	51.732	0.253129
39	newly 3 weeks spec	24.	25.07	{3., 54.}	86.375	0.452806	newly 3 weeks cumul	41.	187.54	{38., 321.}	363.805	0.512905	newly 5 weeks spec	32.	8.71	{0., 20.}	88.0938	1.3353	newly 5 weeks cumul	105.	210.16	{38., 357.}	128.895	0.309152	newly 62 weeks cumul	520.	235.43	{38., 506.}	70.8712	0.59288
40	newly 3 weeks spec	29.	42.5	{1., 105.}	114.069	0.584702	newly 3 weeks cumul	61.	318.95	{19., 835.}	445.59	0.654045	newly 5 weeks spec	48.	28.19	{0., 91.}	86.1458	1.2501	newly 5 weeks cumul	145.	381.46	{24., 836.}	199.366	0.541173	newly 68 weeks cumul	580.	496.94	{38., 907.}	53.0069	0.460795
41	newly 3 weeks spec	22.	53.85	{4., 117.}	182.136	0.512638	newly 3 weeks cumul	38.	526.49	{118., 976.}	1287.18	1.02851	newly 5 weeks spec	81.	16.78	{0., 46.}	81.7284	1.42822	newly 5 weeks cumul	156.	572.28	{125., 1059.}	275.526	0.517682	newly 65 weeks cumul	649.	608.75	{200., 1097.}	48.6641	0.261898
42	newly 3 weeks spec	18.	59.64	{5., 126.}	269.778	0.626784	newly 3 weeks cumul	47.	542.03	{170., 1029.}	1057.98	0.97106	newly 5 weeks spec	26.	18.06	{0., 47.}	87.3077	1.04029	newly 5 weeks cumul	102.	591.73	{222., 1154.}	486.775	0.707352	newly 65 weeks cumul	610.	625.62	{232., 1170.}	50.4492	0.27003
43	newly 3 weeks spec	35.	9.23	{0., 12.}	99.4	1.69633	newly 3 weeks cumul	83.	86.86	{8., 97.}	107.687	0.505759	newly 5 weeks spec	42.	2.49	{0., 4.}	97.0238	2.23089	newly 5 weeks cumul	155.	93.75	{9., 97.}	98.4065	0.713373	newly 58 weeks cumul	514.	98.3	{9., 107.}	91.3969	1.15848
44	newly 3 weeks spec	15.	88.23	{7., 211.}	503.933	0.704126	newly 3 weeks cumul	34.	481.66	{164., 891.}	1322.47	1.06229	newly 5 weeks spec	30.	37.07	{0., 87.}	91.7667	0.663802	newly 5 weeks cumul	77.	574.69	{221., 1054.}	652.377	0.818671	newly 66 weeks cumul	567.	658.75	{262., 1134.}	53.1869	0.255513
45	newly 3 weeks spec	32.	37.31	{0., 71.}	93.4688	0.601783	newly 3 weeks cumul	68.	303.6	{84., 713.}	351.441	0.549334	newly 5 weeks spec	56.	10.65	{0., 27.}	85.8036	1.50122	newly 5 weeks cumul	159.	332.95	{100., 739.}	127.604	0.308342	newly 63 weeks cumul	632.	348.6	{118., 846.}	63.6487	0.466794
46	newly 3 weeks spec	21.	25.94	{3., 57.}	99.7143	0.492221	newly 3 weeks cumul	37.	192.25	{34., 308.}	427.865	0.597804	newly 5 weeks spec	19.	8.87	{0., 27.}	90.6842	1.11262	newly 5 weeks cumul	68.	215.2	{42., 376.}	231.471	0.434378	newly 62 weeks cumul	566.	236.15	{42., 504.}	71.3834	0.640927
47	newly 3 weeks spec	13.	43.67	{1., 115.}	279.923	0.662677	newly 3 weeks cumul	21.	308.32	{19., 818.}	1378.95	0.950855	newly 5 weeks spec	26.	29.8	{0., 99.}	127.308	1.1415	newly 5 weeks cumul	64.	372.07	{42., 826.}	499.078	0.682394	newly 68 weeks cumul	615.	500.99	{42., 916.}	50.8341	0.447117
48	newly 3 weeks spec	37.	32.75	{2., 78.}	88.2432	0.6	newly 3 weeks cumul	81.	230.94	{48., 495.}	202.519	0.370267	newly 5 weeks spec	34.	11.07	{0., 29.}	86.0294	1.3189	newly 5 weeks cumul	153.	259.34	{55., 589.}	111.281	0.313435	newly 62 weeks cumul	483.	287.2	{55., 652.}	69.2836	0.516733
49	newly 3 weeks spec	13.	92.45	{4., 209.}	630.385	0.780454	newly 3 weeks cumul	42.	519.07	{170., 979.}	1141.45	1.00529	newly 5 weeks spec	46.	39.06	{0., 92.}	66.2609	0.720665	newly 5 weeks cumul	113.	615.86	{230., 1090.}	452.69	0.700014	newly 66 weeks cumul	632.	702.45	{293., 1180.}	50.9256	0.261471
50	newly 3 weeks spec	9.	54.17	{3., 120.}	527.222	0.756597	newly 3 weeks cumul	25.	374.28	{148., 834.}	1398.16	1.07614	newly 5 weeks spec	19.	17.19	{0., 37.}	81.2105	0.828877	newly 5 weeks cumul	58.	422.37	{167., 922.}	630.707	0.781794	newly 64 weeks cumul	488.	448.46	{168., 999.}	49.0041	0.25649
